# A Modified Middle Eastern Contrast Sensitivity Chart

**Published:** 2014

**Authors:** Mohammad Bagher Hadavand, Fatemeh Heidary, Roghayeh Heidary, Reza Gharebaghi

**Affiliations:** 1 Health Insurance Organization, Tehran, Iran,; 2 Shiraz University of Medical Sciences, Shiraz, Iran,; 3 Alborz University of Medical Sciences, Karaj, Iran

**Keywords:** Pelli-Robson standard chart, Middle Eastern Characters, Contrast Sensitivity Chart, Glaucoma, Cataract

## Abstract

The contrast sensitivity test has been used to examine vision in different clinical circumstances. Moreover, as contrast sensitivity is affected by several ocular states, its measurement has been considered practically for monitoring and assessment of a wide range of visual functions, predicting vision related abilities, diagnosing several ophthalmic conditions, and evaluating many ocular disorders including glaucoma, cataracts, diabetic retinopathy, optic neuritis and age-related degeneration.

The Pelli-Robson standard chart has been translated and modified using Persian-Arabic characters since illiterates and children in the Middle East, Central Asia, and Africa are more likely to distinguish Arabic characters instead of English ones. The translation of these characters is expected to have more precise results, thereby improving the test’s validity and provide early diagnoses of ocular problems. This manuscript is focused on conceptions relating to the project. Further studies are required to evaluate the sensitivity, specificity, and reliability of the revised chart to best compare it to the standard Pelli-Robson one.

## INTRODUCTION

Arabic is used by 22 members of the United Nations Educational, Scientific and Cultural Organization (UNESCO), and is the language of a population of more than 422 million in the world. More than 1.5 billion Muslims worldwide also use Arabic since it is the language of the Islam religion (1). Additionally, a significant number of people in Iran, Pakistan, Turkey, Bangladesh, Indonesia, and Malaysia may find it easier to read Arabic letters and scripts than English.

 Although there has been a trend towards localizing standard charts for specific regions, very few studies are available regarding the design of visual charts based on Arabic letters to help illiterates and children living in these regions in particular (2-5).

 Considerable research has proven an association between contrast sensitivity, abilities and tasks related to daily human life such as mobility, face recognition, driving, computer task accuracy, and reading speed. Additionally, many other studies have indicated the beneficial effects of contrast measurement in the early diagnosis of ocular disorders as well as in monitoring their progressionsuch as glaucoma, cataracts, diabetic retinopathy, optic neuritis and age-related degeneration. Contrast measurement is also of demonstrated value in evaluating post-cataract surgery, laser capsulotomy, intraocular lenses, laser photocoagulation, contact lens users, radiation therapy, and finally, refractive laser surgeries. Therefore, measurement of contrast sensitivity is of great clinical importance (6,7).

 Many charts have been used to evaluate contrast sensitivity, but the Pelli-Robson is the standout with its reliability and ease of use (as clinically proven by many researchers), and it has been found that it is able to detect many defects in visual performance in patients with a wide variety of ocular disorders (8). Therefore, the design of our chart to assess the contrast sensitivity was based on the standard Pelli-Robson chart.

## HYPOTHESES/IDEA

Our goal was to design a new chart according to the routinely used Pelli-Robson based on Persian-Arabic characters. The standard chart was translated from English and modified to assist illiterates as well as children located in the region.

 According to the basics of the Pelli-Robson chart (9), our chart was placed at 1 meter, and the subject was to wear a baseline of an additional +0.75 DS refractive correction.

**Figure 1:**
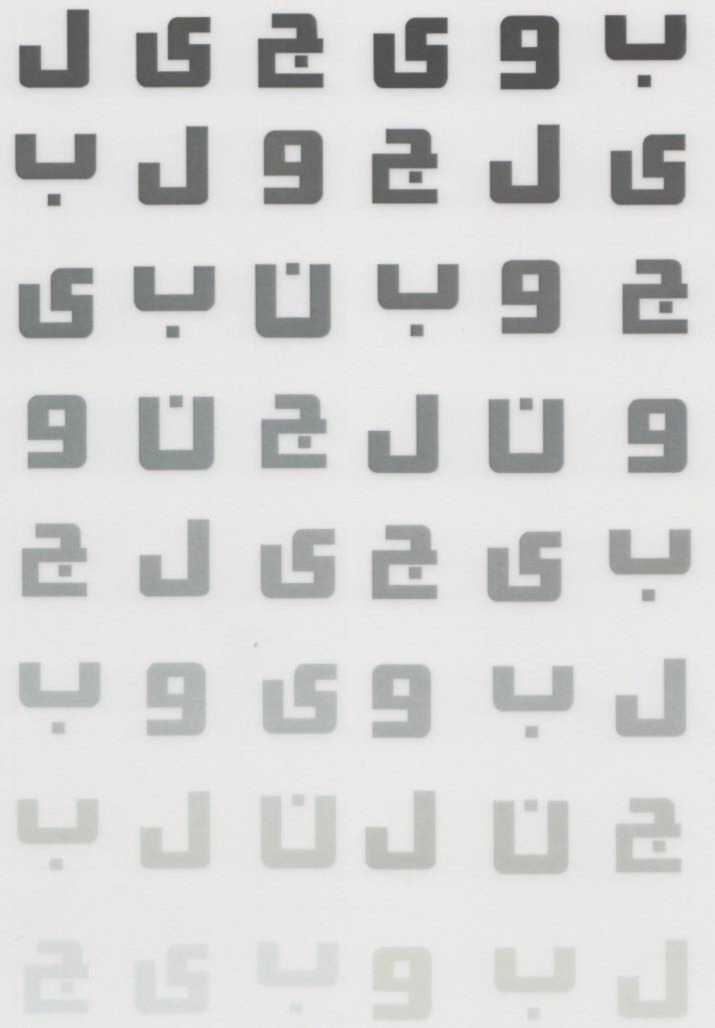
Scanned copy of a modified Middle Eastern contrast sensitivity chart.

 The chart’s size is 59 to 84 cm, printed on resin coated paper containing 16 triplets of Sloan letters, which are arranged in 8 lines, with each line having 2 sets of triplets all of invariable size (Figure 1).

 For determine contrast, each line contains 3 letters, and all the letters in a similar group have some contrast, but the top row with 3 letters has the highest contrast, and the bottom row has the lowest. This trend decreases for each consecutive group of three letters. 

 To assess the patient’s contrast sensitivity, the last group in which the patient is able to read 2 out of 3 letters was scored. Like the standard Pelli-Robson chart, scoring sheets were prepared (10). Here we used a letter-by-letter scoring system, and each correctly identified letter was scored as 0.05 log, except for the first triplet in which the contrast was considered as 100%. Patients were asked to read from the top letters coming down. Though they were asked not to move, head movements were permitted.

 The examination was conducted in a quiet room, with the illumination the same as is standard when testing with the Pelli-Robson chart. To avoid any shadows in the room, no local source of lighting was used. For monocular assessment of contrast sensitivity, the patient’s right or left eyes were occluded with the palm of their hands, and their vision was evaluated by using appropriate correction of refractive error.

## DISCUSSION

Pelli-Robson chart has been used by many investigators worldwide for many years, but its acceptance through clinicians has been fairly low (7). Contrast sensitivity is as important as visual acuity to assess visual performance. Contrast sensitivity measurement helps to distinguish objects that differ in size at low contrast (11).

 Our modified test was designed based on Persian-Arabic characters for measuring contrast sensitivity in illiterates and children in regions where Arabic characters were more common than English. We did not compare this modified test with the standard charts in the same population, so we recommend comparison of the new chart with the standard chart (especially Pelli-Robson) to better evaluate the new chart’s sensitivity, specificity, and repeatability.

 Several criteria must be achieved for a new test to be used as a routine clinical test for screening. One of the most important is the test reliability, therefore assessing the similarities between the new test and the standard one seems essential (12). 

 This manuscript's only focus is strictly about the idea and design of the new test, therefore further investigation is required for the evaluation of the new chart. We also suggest that computer tests are designed to be performed easily on a LCD monitor, as well as new handheld charts, which could be used at a working distance with the same optotypes.
